# Successful treatment of a refractory intestinal Behcet’s disease with an oncology history by Vedolizumab: a case report and literature review

**DOI:** 10.3389/fimmu.2023.1205046

**Published:** 2023-05-23

**Authors:** Ruixia Li, Xiaofei Li, He Zhou, Yanting Shi, Fang Wang, Tong Wu, Jie Liang

**Affiliations:** State Key Laboratory of Cancer Biology, National Clinical Research Center for Digestive Diseases and Xijing Hospital of Digestive Diseases, Air Force Military Medical University, Xi’an, China

**Keywords:** Behcet’s disease, intestinal disease, vedolizumab, tumor, case report

## Abstract

**Objective:**

Behçet’s Disease (BD) is an intractable systemic vasculitis. When accompanied by intestinal symptoms, the prognosis is usually poor. 5-Aminosalicylic acid (5-ASA), corticosteroids, immunosuppressive drugs, and anti-tumor necrosis factor-α (anti-TNF-α) biologics are standard therapies to induce or maintain remission for intestinal BD. However, they might not be effective in refractory cases. Safety should also be considered when patients have an oncology history. Regarding the pathogenesis of intestinal BD and the specific targeting effect of vedolizumab (VDZ) on the inflammation of the ileum tract, previous case reports suggested that VDZ might be a potential treatment for refractory intestinal BD.

**Methods:**

We report a 50-year-old woman patient with intestinal BD who had oral and genital ulcers, joint pain, and intestinal involvement for about 20 years. The patient responds well to anti-TNF-α biologics but not to conventional drugs. However, biologics treatment was discontinued due to the occurrence of colon cancer.

**Results:**

VDZ was intravenously administered at a dose of 300 mg at 0, 2, and 6 weeks and then every eight weeks. At the 6-month follow-up, the patient reported significant improvement in abdominal pain and arthralgia. We observed complete healing of intestinal mucosal ulcers under endoscopy. However, her oral and vulvar ulcers remained unresolved, which disappeared after adding thalidomide.

**Conclusion:**

VDZ may be a safe and effective option for refractory intestinal BD patients who do not respond well to conventional treatments, especially those with an oncology history.

## 1 Introduction

Behçet’s Disease (BD) is systemic vasculitis that is usually refractory, mainly characterized by ulcers (oral and genital) and lesions (ocular and skin), which may be accompanied by joint, intestinal, neurological, and vascular involvement (1). The intestinal BD morbidity rate ranges from 5% to 20% globally, and it is more common in the Mediterranean region and East Asian countries, especially in South Korea, Japan, and China (2). When patients with BD present with abdominal pain, diarrhea, bloody stools, and abdominal masses, an endoscopy should be performed as soon as possible to confirm the diagnosis. It is also essential to distinguish it from inflammatory bowel disease (IBD), intestinal tuberculosis, or infectious enteritis, which may also present with the above non-specific intestinal symptoms (3). In the recent guidelines (1), anti-TNF-α biologics are recommended for refractory cases that do not respond to conventional drugs, including 5-ASA, corticosteroids, and immunosuppressive drugs. However, some patients continue to respond poorly due to limitations such as primary or secondary loss of response, intolerance, or contraindications (4). TNF-α also plays a vital role in apoptosis and tumor suppression, and interference with relative pathways may increase the risk of malignancy (5). Therefore, it is necessary to consider other alternative treatments for refractory intestinal BD patients with an oncology history.

Although the pathogenesis is unknown, many factors are thought to contribute to BD, including genetics, environment, infection, microbiota, and immune status (6–9). Imbalance in the numbers of T cells (especially Th1, Th2, and Th17), natural killer cells, and inflammatory cytokines play an essential role (10, 11). The elevated levels of IL-1β, IL-6 and TNF-α in the gastrointestinal mucosa of patients with intestinal BD play a pathogenic role in the increasing inflammatory responses (12). VDZ can specifically target α4β7 integrins expressed on the surface of intestinal lymphocytes, preventing the recruitment of pro-inflammatory cells to the intestine from reducing inflammation (13–15).

As far as we know, only one report of VDZ being used in patients with intestinal BD (16). Six months after the infusion of VDZ, the patient achieved clinical remission. Here we continue to report a case of an intestinal BD patient with an insufficient response to conventional therapy who achieved a better outcome after conversion to VDZ. We also discuss the safety of VDZ in patients with a combined oncologic history.

## 2 Case report

**Figure 1:**
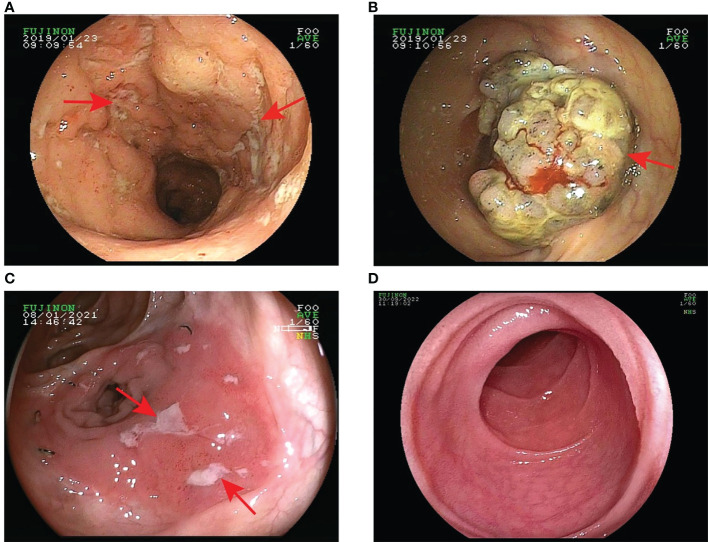
Changes in the patient’s intestinal mucosa. **(A)** In January 2019, prior to the application of infliximab, colonoscopy showed multiple ulcers at the end of the ileum; **(B)** In August 2019, colonoscopy revealed a large bulging lesion in the cecum and ascending colon; **(C)** In January 2021, colonoscopy again showed multiple large ulcers in the ileum and anastomosis; **(D)** In September 2022, one year after vedolizumab treatment colonoscopy showed complete healing of the ileal ulcers.

**Figure 2:**
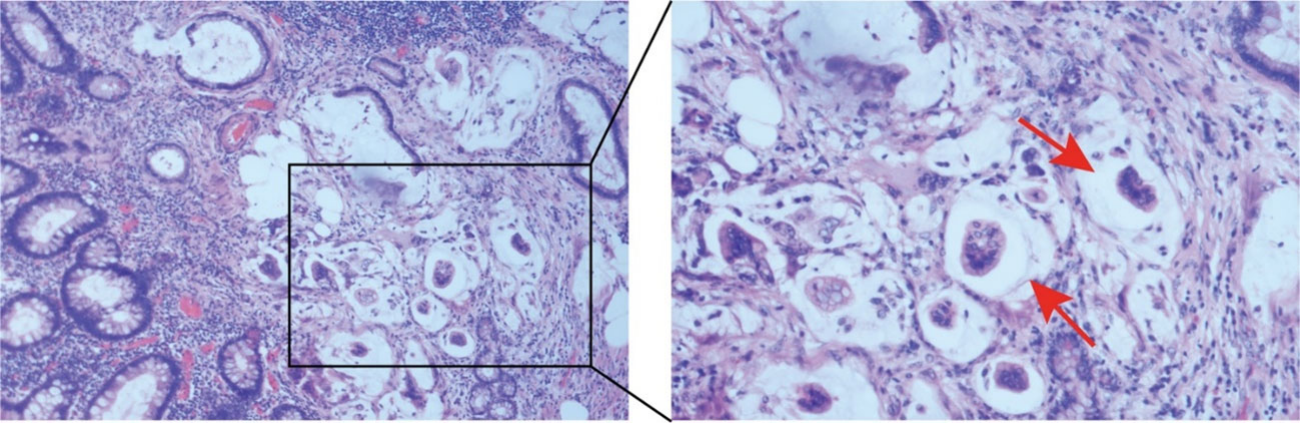
Pathology images from the right radical hemicolectomy. H&E staining revealed an irregular glandular arrangement of the mucosal epithelium with a large amount of mucus visible in the background and infiltrative growth.

We here describe a 50-year-old female patient with intestinal BD. The clinical characteristics and treatment measures of the patient are shown in **Table 1**. The patient has had recurrent oral and vulvar ulcers since 2003. In March 2004, she developed nocturnal hyperthermia and dark red bloody stools. Colonoscopy showed terminal ileum ulcers and proliferative lesions. Pathological biopsies reported acute and chronic inflammation of the superficial mucosa. Antinuclear antibody (ANA) and pathergy test results were positive, and the pure protein derivative (PPD) test was negative. Indexes such as infection, tumor, and autoantibody series were normal. According to the diagnostic criteria of the International Study Group for Behçet’s Disease (17), and combined with clinical symptoms, laboratory tests, colonoscopy, and pathological findings, the patient was diagnosed with BD. Prednisone (40mg PO daily), cyclophosphamide (50mg PO daily), and mesalazine (4g PO daily) were administered to control symptoms. Prednisone and cyclophosphamide decreased gradually. In December 2005, cyclophosphamide was stopped, and 5mg of prednisone was taken orally daily to maintain symptoms. In August 2007, she developed multiple joint pains. There were no abnormalities in Anti-Streptolysin “O” (ASO), rheumatoid factor (RF), human leukocyte antigen (HLA-B27), and anti-neutrophil cytoplasmic antibody (ANCA). The treatment regimens were adjusted repeatedly according to the patient’s clinical symptoms, including prednisone (40mg PO daily), sulfasalazine (3.0mg PO daily), methotrexate (12.5mg PO weekly), thalidomide (100mg PO daily), cyclophosphamide (50mg PO daily) and colchicine (1mg PO daily), etc. In January 2019, the patient’s condition worsened with persistent right lower abdominal pain, bloody stools, frequent episodes of oral ulcers, and arthralgia. Colonoscopy showed multiple ulcers in the distal ileum and large protuberant lesions in the cecum and ascending colon (**Figures 1A, B**). The biopsies revealed chronic inflammatory activity of the mucosa with ulcer formation, and no evidence of neoplasia was seen. Considering the patient’s recurrent illness, no significant improvement in intestinal mucosal status, and the failure of previous medications, we decided to switch to treatment with anti-TNF-α biologics —– infliximab (IFX). We truthfully stated the benefits and risks of using biologics and obtained the informed consent of the patient. IFX was administered with a dose of 300 mg at 0, 2, 6 weeks, and then every eight weeks. The patient reported a significant reduction in oral and vulvar ulcers, abdominal pain, and arthralgia immediately after the first infusion. On August 9, 2019, a colonoscopy showed that the terminal ileal ulcer was better than before. However, there were no significant changes in ileocecal and ascending colon masses. Biopsies revealed acute mucous inflammation, small vessel dilatation and congestion, epithelial hyperplasia on the recessed surface with erosion and inflammatory exudation, and significant glandular hyperplasia and mucus secretion. There was no evidence of granuloma or neoplasia. Intestinal dual-source CT showed obvious thickening at the lower end of ascending colon, soft tissue shadow protruding into the lumen, and abundant blood supply. Endoscopists considered performing endoscopic submucosal dissection (ESD) to be risky. The gastrointestinal surgeon reported that the possibility of colon cancer could not be excluded from the ascending colonic mass and recommended surgical excision to determine its nature. Therefore, after multidisciplinary discussions, the patient underwent a right radical hemicolectomy on September 6, 2019. Post-operative pathology confirmed the mucinous tumor (**Figure 2**). IFX was suspended. The mFOLFOX6 chemotherapy regimen was administered for four cycles before being discontinued due to the global outbreak of the novel coronavirus. Long-term oral thalidomide (50mg PO daily) to control the disease. In January 2021, the patient’s right lower abdominal pain worsened again. Colonoscopy showed multiple large ulcers in the ileum and anastomotic orifice (**Figure 1C**). Enhanced CT of the abdomen showed thickening of the anastomotic wall and the proximal ileal wall of the anastomosis with significant enhancement.

**Table 1:** Clinical characteristics and therapeutic interventions of the patient.

Items	
Sex	F
Age(yrs)	50
Intestinal BD course(yrs)	19
Clinical features	Oral ulcers, genital ulcers, Joint pain, intestinal symptoms and ulcers (terminal ileum)
Previous treatment	CS, CTX, IFX, MTX, SASP, THD, Colchicine, Mesalazine
VDZ combined therapies	THD
Present therapies	VDZ 300 mg every 8 weeks
Follow-up(mths)	16
Clinical response	success

BD, Behcet’s Disease; CS, corticosteroids; CTX, cyclophosphamide; IFX, infliximab; MTX, methotrexate; SASP, salazosulfapyridine; THD, thalidomide; VDZ, vedolizumab.

**Figure 3:**
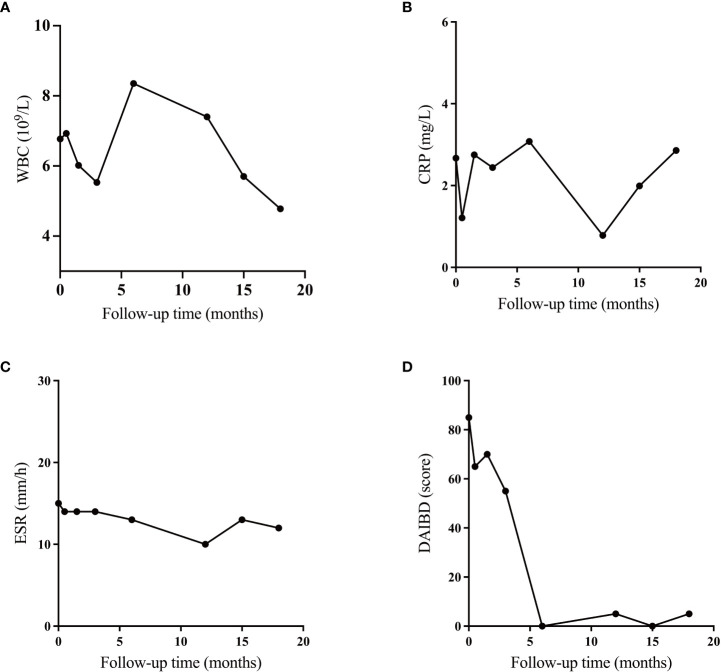
Efficacy of vedolizumab in the treatment of intestinal Behcet’s Disease. **(A)** Changes of white blood cell (WBC); **(B)** Changes of C-reactive protein (CRP); **(B)** Changes in erythrocyte sedimentation rate (ESR); **(C)** Changes in indices of disease activity in intestinal Behcet’s Disease (DAIBD).

The intestinal BD recurred. The immunosuppressive effect of anti-TNF-α biologics may be risky for patients with an oncology history. Therefore, combined with the pathogenesis of intestinal BD and previous case reports, we decided to try VDZ for some clinical benefits. On October 9, 2021, the patient received an infusion of 300 mg VDZ (initially at 0, 2, and 6 weeks, then every eight weeks). After a short follow-up of 6 months, the patient reported that her lower abdominal pain and arthralgia were improved. No obvious adverse reactions occurred. The DAIBD score decreased from 85 to 0, and the WBC, ESR, and CRP levels were normal. On September 30, 2022, a colonoscopy showed a completely healed ileal ulcer with a smooth mucosal surface and well-dilated intestine (**Figure 1D**). However, at the 15th monthly follow-up, her oral and vulvar ulcers recurred, and the CRP concentration increased. Therefore, thalidomide was administered to alleviate systemic inflammation. As shown in **Figures 3A–C**, although all systemic inflammatory indices showed repeat increases, such as WBC, CRP, and ESR, these might be caused by the presence of inflammation outside of the gastrointestinal (GI) tract. As only focused on the GI tract (**Figure 3D**), the DAIBD showed the significant efficacy of VDZ on intestinal BD.

## 3 Discussion and literature review

We report here a case of a refractory intestinal BD patient. She had recurrent oral and genital ulcers, joint pain, and intestinal involvement for nearly 20 years. Anti-TNF-α biologics relieved symptoms when initially applied. However, the patient developed colon cancer during BD. We performed surgical excision and chemotherapy intervention. During follow-up, her intestinal BD recurred. Considering the aggravation of the intestinal condition and the combined tumor history, we decided to try VDZ. At the 6-month follow-up, she achieved clinical remission, all laboratory test results were in normal ranges, and mucosal healing was observed under endoscopy. Several studies have demonstrated that VDZ are effective on extraintestinal manifestations of IBD (18, 19). However, oral and vulvar ulcers still occurred sporadically in the patient we report here, and the symptoms disappeared with the combination of thalidomide.

Unlike other gastrointestinal inflammatory diseases, which are primarily chronic and persistent, BD is a recurrent acute systemic vasculitis (20). Different clinical manifestations can occur individually or coexist in the same patient (21). Typical geographic distribution, infection, immunization, and environmental factors may contribute to the development of BD (10). Vascular damage, neutrophil hyperfunction, and autoimmune reactions are the key characteristics of BD. Analysis of HLA phenotype and serum IgD levels may help to make a diagnosis (20).

Intestinal involvement in BD is usually associated with poor prognosis and a risk of severe organ damage or even death. The spectrum can involve any digestive organ from the oral cavity to the anus, especially the ileocecal region, transverse colon, and ascending colon (22). In Asian countries, ileocecal involvement appears to be more common (23), and volcano type ulcers are typical endoscopic manifestations (1). Endoscopy, computed tomography, and magnetic resonance enterography are used to assess intestinal involvement and disease activity in BD (24).

The ultimate treatment goals for patients with intestinal BD are the disappearance of clinical symptoms, normalization of inflammatory indices, and intestinal mucosal healing (1). The acute phase is usually treated with corticosteroids combined with 5-ASA or azathioprine (AZA) (3). Alternative treatment options include salazosulfapyridine (SASP), mycophenolate mofetil (MMF), and methotrexate (MTX) (25). Patients with severe or refractory intestinal symptoms should be treated with anti-TNF-α biologics alone or in combination with thalidomide (THD) (1). Anti-TNF-α biologics have demonstrated promising results in inducing and maintaining remission of intestinal BD (26, 27). However, due to the role of TNF-α in NK cell- and CD8+ T cell-mediated clearance of tumor cells (28), it can trigger apoptosis through an exogenous pathway by activating caspases 8 and 10. It may also activate signaling through the NF-κB pathway, which indicates anti-TNF-α biologics may promote tumor recurrence, growth, and/or metastasis (5, 29, 30).

The patient we report here had multiple colonic mucosal biopsies, all of which indicated inflammatory lesions. In 2019, we again performed large biopsies of the ileal and ascending colon masses prior to the use of IFX. Neoplastic lesions were still excluded, suggesting chronic mucosal inflammatory activity. We obtained the patient’s informed consent for the use of IFX and the symptoms remitted. However, colonoscopy showed no significant changes in the masses. After multidisciplinary discussion, we decided to surgically resect the hyperplastic lesions, and postoperative pathology revealed local tumor. It cannot be absolutely excluding that the appearance of cancerous lesions is due to the use of IFX. However, prior to the use of IFX, we have repeatedly performed pathological biopsies of the masses to exclude malignant lesions. The current reports on anti-TNF-α biologics and tumors are mainly on skin cancers, including melanoma (31–34). ECCO guidelines recommend that infliximab is best avoided in patients with a history of malignancy (35). Therefore, it is necessary to consider other alternative therapies for patients with refractory intestinal BD with an oncology history.

Our literature review found a case report of VDZ successfully treating refractory BD (16). The patient had erythema nodosum, oro-genital ulcers, and biopsy-proven intestinal BD, which was not successfully treated with conventional immunosuppressants and several biologics agents, including anti-TNF-α biologics. Based on previous reports of the efficacy of VDZ in IBD, the authors decided to administer VDZ to treat severe intestinal involvement. The results led to a satisfactory gastrointestinal response and the concomitant disappearance of ulcerations, arthralgia, and a reversion of the skin lesions. VDZ, an intestine-selective humanized monoclonal IgG1 antibody, has been approved for treating IBD (36, 37). VDZ blocks the recruitment of pro-inflammatory cells and dendritic cells to the inflamed gut by specifically targeting α4β7 integrins expressed on the surface of intestinal lymphocytes and monocytes and inhibiting their binding to cell adhesion molecules (CAMs) (15, 38, 39), leading to alterations in the innate and acquired immune cell program to suppress inflammation without interfering with transit to other organs (40). Compared to anti-TNF-α biologics or other immunosuppressive agents, integrin receptor antagonists reduce the side effects associated with systemic immunosuppression. Moreover, studies reported no increase in malignancy and mortality among VDZ-exposed patients (41).

Growing evidence shows that IBD and BD may be closely related and are part of a typical disease spectrum (42). They have similarities in plausible pathophysiological features. We should note that VDZ had a good response in the ileum portion and not in the whole tract of intestine. That in IBD there is a heterogeneous distribution of immune cells in the enteric tract, with the presence of CD4+ memory (mem), lymphocytes, B and dendritic cells in the ileum (43). The most α4β7 positive cells are CD4+mem, CD8+mem and B cells, which can explain not only as to why VDZ works better on lesions in some parts of the intestine (as ileum) and less so in others (44), but also as too why VDZ did not work on ulcer in the other mucous as oral and vulval ones. This is probably due to the heterogeneous distribution of the α4β7 positive cells in these mucous.

The relationship between IBD and colorectal cancer has been demonstrated (45). Although BD is clinically similar to IBD. Only a tiny series of tumor-related cases had been reported, of which colon cancer is less common (46–48). There is no clear evidence that BD causes epithelial cancer or sarcoma. The patient we reported was diagnosed with colonic mucinous carcinoma 16 years after the first presentation of BD symptoms. The severity and duration of inflammation may be risk factors for cancer (45, 49). As increasing numbers of potent drugs are used to treat BD, patients may live longer than before and therefore be more likely to develop the malignant disease. In addition, extensive vasculitis and abnormal immune regulation in BD are thought to be mechanisms of increased risk of malignancy (50). Further detailed pathological studies, cytogenetic analysis, and long-term sizeable prospective cohort studies are needed to clarify the relationship between BD and tumors.

## 4 Conclusion

VDZ specifically blocks T-cell chemotaxis to the ileum during inflammation and inhibits inflammatory factor signaling. It is suggested that it may serve as a potential treatment drug for intestinal BD, especially involving the ileum tract. The patients with intestinal BD we reported here had poor efficacy to conventional drugs. Anti-TNF-α biologics had shown better therapeutic effects. However, due to the occurrence of a colon tumor, we attempted to switch to VDZ. The intestinal symptoms and joint pain were reduced, and intestinal mucosa ulcers healed completely. No recurrence or other side effects occurred. Thus, our example provided possible evidence for the efficacy and safety of VDZ in patients with refractory intestinal BD who have an oncology history. As an intestinal-specific antibody, VDZ has shown an inhibitory effect on ileal inflammation but is less effective in treating systemic vasculitis. During the follow-ups, the manifestations like oral and vulvar ulcers and increased CRP were further resolved by thalidomide.

Short follow-up time and only one case reported are our major limitations. Large and long-term clinical trials are needed to verify the efficacy and safety of VDZ in treating intestinal BD. In addition, in the intestinal BD the effect and mechanism of VDZ on the other tracts of intestine and on other mucous membranes need to be further explored.

## Data availability statement

The original contributions presented in the study are included in the article/supplementary material. Further inquiries can be directed to the corresponding authors.

## Ethics statement

Written informed consent was obtained from the individual(s) for the publication of any potentially identifiable images or data included in this article.

## Author contributions

TW and JL contributed to conception and design of the study. RL organized the database. XL performed the statistical analysis. RL, XL and HZ wrote the main content of the manuscript. YS and FW provided tables and pictures. All authors contributed to the article and approved the submitted version.
